# Discrimination Between Normal and Cancerous Cells Using AFM

**DOI:** 10.1007/s12668-016-0191-3

**Published:** 2016-01-30

**Authors:** Małgorzata Lekka

**Affiliations:** Institute of Nuclear Physics, PAS, Radzikowskiego 152, 31-342 Kraków, Poland

**Keywords:** Atomic force microscopy, Cancer cell detection, Mechanosensing, Cancer cell elasticity

## Abstract

Currently, biomechanics of living cells is in the focus of interest due to noticeable capability of such techniques like atomic force microscopy (AFM) to probe cellular properties at the single cell level directly on living cells. The research carried out, so far, delivered data showing, on the one hand, the use of cellular mechanics as a biomarker of various pathological changes, which, on the other hand, reveal relative nature of biomechanics. In the AFM, the elastic properties of living cells are delivered from indentation experiments and described quantitatively by Young’s modulus defined here as a measure of cellular deformability. Here, the AFM studies directly comparing the mechanical properties of normal and cancerous cells are summarized and presented together with a few important issues related to the relativeness of Young’s modulus.

## Introduction

Oncogenic transformation leads to a distinct phenotype of cancerous cells in such aspects such as variations in cellular growth, differentiation and interactions with neighbouring cells and/or the extracellular matrix (ECM) and also in internal structure and properties of single cells [1, 2]. Among others, alterations in the structure and organization of cytoskeleton manifest, in most cases, in larger deformability of single cells, as it has been reported for various cancers, such as bladder, prostate, thyroid and ovarian ones [3–8]. Furthermore, these changes are commonly related to either a partial loss of actin filaments [9] or disorganization of microtubules [10] being in fact the consequence of lower density of the cellular scaffold. The deformability of single cells has been studied for a long time using various techniques. The driving force for such studies is the assumption that, depending on the disease type, the altered cellular deformability (or lack of it) should play a critical role in the development and progression of various diseases. Thus, it can be a manifestation of diseases with genetic mutations linked with structural/molecular changes on a single cell level observed, for example, in various muscular dystrophies [11–13] or laminopathies [14, 15]. The increased/decreased deformability manifests in various cancers. This observation attracted researchers from the fields of cellular biology, biophysics and medicine due the potential ability to define a non-labelled biomarker of cancer progression.

The technological development towards measurements of individual cells at the single cell level has brought powerful techniques that can bind and relate the mechanical properties with cellular functioning and structures. One of such techniques is the atomic force microscopy (AFM) [16]. The pioneering studies showed the importance of mechanical properties to characterize cancerous cells [3]. In these studies, the deformability of human bladder cancerous cells was 1 order of magnitude larger as compared to reference cells originating from non-malignant cancers. These early results have been supported (and indirectly verified) by optical tweezers measurements. Using this high-throughput technique, three cell lines were compared, namely a non-tumorigenic breast epithelial MCF10 cells; a non-motile, non-metastatic breast epithelial cancer MCF7 cells; and MCF7 cells transformed with phorbol ester. The results showed a significant increase in deformability in more invasive cells (i.e. transformed MCF7 breast cells) as compared to both non-metastatic MCF10 and non-transformed MCF7 ones [4]. Further development in the single cell elasticity measurements and data analysis delivered a large database of cases showing significantly larger deformability of single cancerous cells [5, 6, 17]. In the AFM, the deformability is expressed by Young’s modulus that delivers a quantitative measure of cellular elastic properties. It has been reported that cells in vitro have the Young’s modulus values in the range of 1–100 kPa [18]. These values characterize different types of investigated cells, including vascular smooth muscle cells, fibroblasts, bladder cells, red blood cells, platelets and epithelial cells, both normal and cancerous ones.

In the presented work, I would only like to focus on papers showing a direct comparison of mechanical properties between normal and cancerous cells that were measured using AFM. Thus, the brief introduction to atomic force microscopy, the essential technical aspects of elasticity measurements together with exemplary results, is discussed.

## Atomic Force Microscopy

**Fig. 1:**
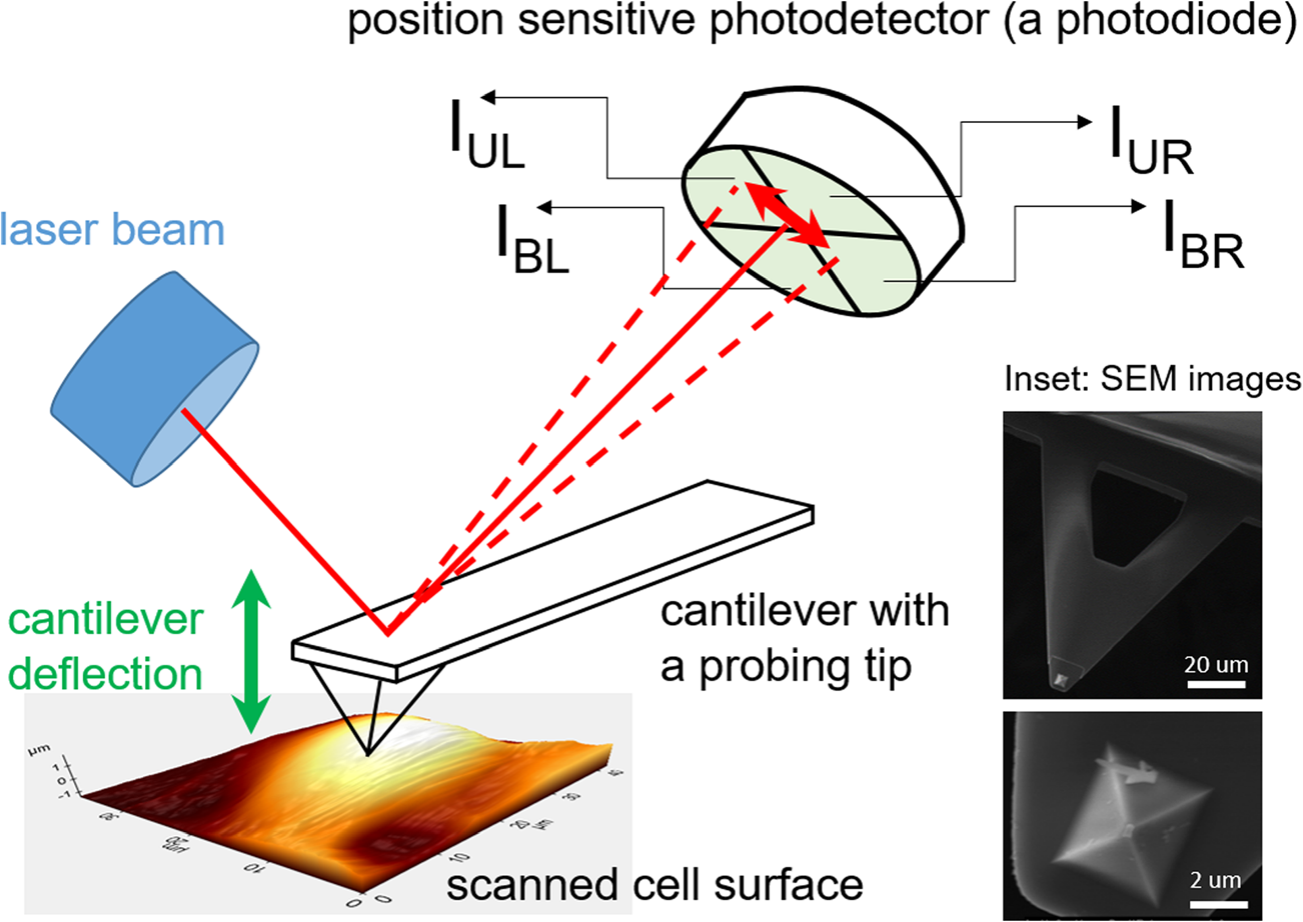
The idea of the atomic force microscopy (AFM). *Inset* shows images of the cantilever (MLCT) obtained from scanning electron microscopy (SEM)

The construction of the atomic force microscope (AFM) [16] can be divided into three main parts, namely a cantilever, a system that detects its deflection and a system that enables scanning and positioning (Fig. 1). The principle of operation is independent of the environment surrounding the cantilever (air, vacuum or liquids). The scanning and positioning system utilizes piezoelectric materials, enabling, on the one hand, a very accurate positioning and scanning and, on the other hand, convenient sample handling and mounting. The key part of the AFM is a cantilever. Cantilevers are lithographically etched from silicon or silicon nitride in a form of long, flexible levers (rectangular or triangular ones), with a probing tip mounted at their free ends (inset in Fig. 1, showing a four-sided pyramid attached at the end of a triangular cantilever made of silicon nitride–cantilever type MLCT, Veeco). Mechanical properties of a cantilever are characterized by the corresponding spring constants which, in case of living cell studies, typically ranges from 0.01 to 0.1 N/m. Often, a probing tip has a shape of a four-sided pyramid (as shown in the inset in Fig. 1) but other geometries, such as cones or spheres, are also used in the AFM experiments. The choice of appropriate geometry of the probing cantilever depends on the type of experiments to be carried out. In the case of elasticity measurements of living cells, the more blunt tips are better since they do not induce large pressure within the contact area between the probing tip and cell surface. The majority of the results showing the differences between normal and cancerous cells have been obtained with the use of pyramidal tips. However, spheres have been also applied, since such geometry of probing tip fulfils better the Hertz model assumptions commonly used in the Young’s modulus value estimation.

The forces acting between the probing tip and a sample (here, a living cell) surface cause the cantilever deflection. The most frequent way of its detection uses the optical system composed of a laser and a photodetector. In such system, the laser beam is focused at the free end of the cantilever just above a probing tip. The reflected beam is guided towards the centre of the photodiode, a position-sensitive detector, whose active area is divided into four quadrants. When the cantilever’s probing tip is far away from the surface, the cantilever is not deflected from its initial position, while the reflected laser beam is set in such a way that photocurrents from each quadrant have similar values. When interacting forces deflect the cantilever, the position of the reflected laser beam changes, leading to different values of photocurrents recorded in the quadrants. If the cantilever bends vertically (i.e. perpendicular to the investigated surface that relates to a force acting perpendicularly to the surface), by appropriate summation and subtraction of the photocurrents, the cantilever normal deflection (ND) can be obtained as follows:

1 $$ \mathrm{N}\mathrm{D}\ \left(\mathrm{V}\right)=A\cdot \left[\left({I}_{\mathrm{UL}}+{I}_{\mathrm{UR}}\right)-\left({I}_{\mathrm{BL}}+{I}_{\mathrm{BR}}\right)\right] $$

where *A* is the proportional coefficient and *I*_*xy*_ is the single quadrant current (U = up, B = bottom, L = left, R = right). In many devices, the deflection is normalized by dividing (1) by the total value of photocurrent from all quadrants. This operation minimizes the effect of power laser fluctuations. Cantilever twists, related to forces acting laterally to the investigated surface, will not be considered here as they reflect friction forces.

Knowing the mechanical properties of the cantilever (i.e. its spring constant *k*_C_), the interaction force can be obtained by multiplying the D by the *k*_C_ value and by the photodetector sensitivity (*S*)

2 $$ F\ \left(\mathrm{n}\mathrm{N}\right)=\mathrm{D}\kern0.5em \left(\mathrm{V}\right)\cdot {k}_{\mathrm{C}}\ \left(\mathrm{N}/\mathrm{m}\right)\cdot S\ \left(\mathrm{n}\mathrm{m}/\mathrm{V}\right) $$

**Fig. 2:**
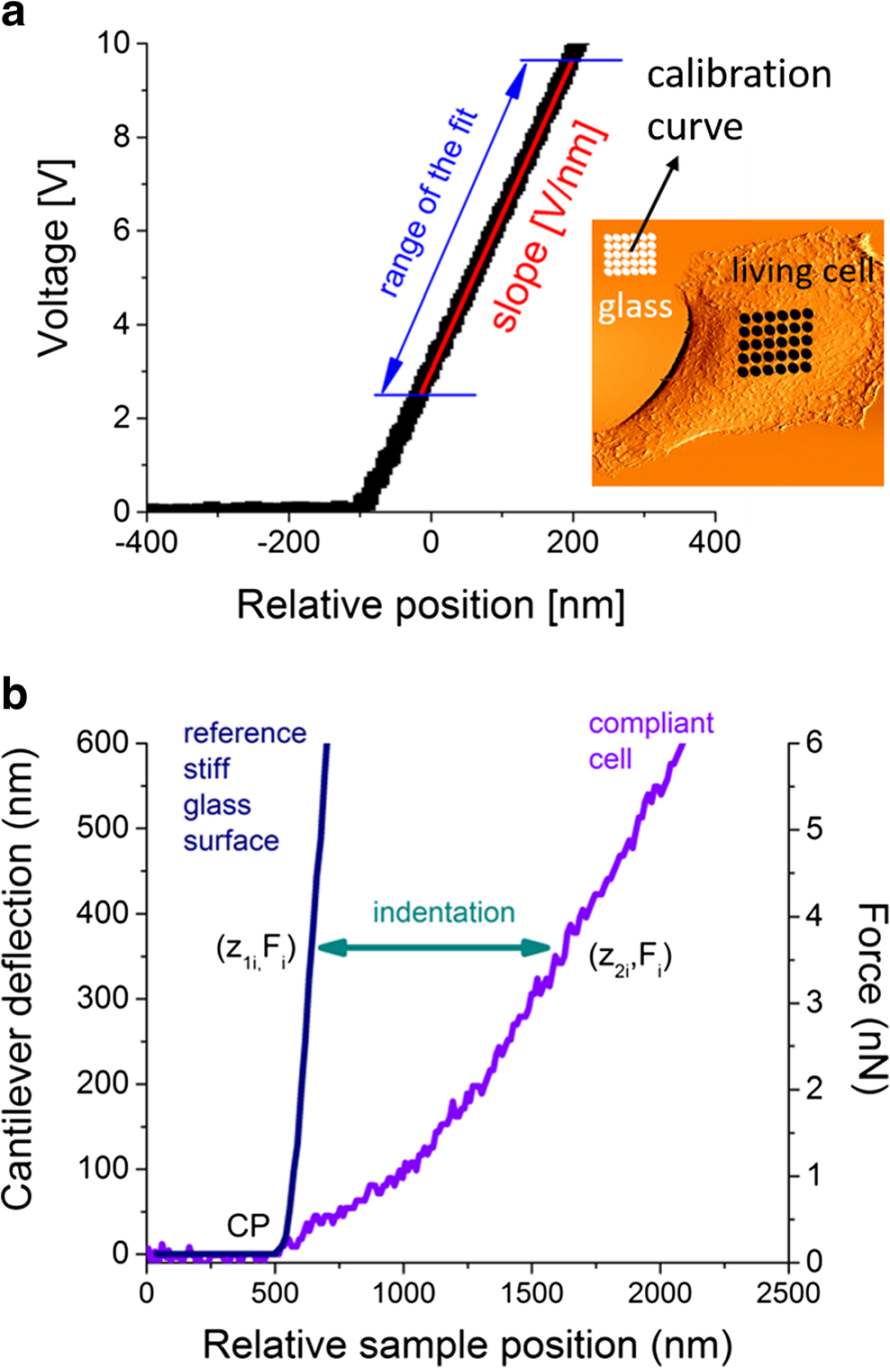
**a** The illustration of the experimental way of AFM elasticity measurement on a living cell, **b** the determination of force versus indentation curve and the resulting force–indentation curve used for the Hertz model fit

The photodetector sensitivity (*S*) relates the deflection of the cantilever (measured in volts) to its deflection in nanometres. To obtain this value, it is important to calibrate the AFM directly before the elasticity measurements. Typically, the calibration is realized by preparing a sample with cells at the density assuring empty spaces between them. The uncovered glass surface is infinitesimally stiff for the load forces applied in the AFM; so, it can be treated as a reference, calibration surface (Fig. 2a). The force curve (i.e. a relation between the cantilever deflection and a relative position of a sample or cantilever) recorded on a stiff surface consists of two parts: horizontal and sloped. The horizontal one describes the situation when the cantilever is away from the investigated surface and the interacting forces are negligible. In such conditions, cantilever deflection oscillates around zero. The slopped line shows the relation between the cantilever deflection and the scanner displacement. A proportionality factor delivers the conversion factor from volts to nanometres, which is an inverse of the photodetector sensitivity. The practical realization of the calibration protocol sets a grid of points over the glass surface, in which individual force curves are acquired (white dots in the topography image presented in Fig. 2a). These measurements deliver an average sensitivity value and determine its uncertainty. Once device is calibrated, the measurements of elasticity properties of a cell can be carried out. Typically, they are realized in the similar manner, by setting a grid of points placed, usually, over a central part of the studied single cell (black dots in the topography image presented in Fig. 2a) previously localized either by optical or AFM topography images. The other experimental parameters, such as approach speed related to load rate, scan area and density of points, are chosen in accordance with the experimental needs and aims.

The elastic properties of living cells are determined by analyzing force versus indentation curves that are not directly measured. A single indentation curve is derived by subtracting the reference deflection (measured on hard, non-deformable surface) from that recorded on a compliant cell (Fig. 2b).

For stiff, non-deformable materials, the cantilever deflection is proportional to the relative position of the sample while, for compliant materials, the deflections are smaller and the resulting curve has a non-linear character. The difference between these curves determines the deformation of the sample surface. For a given load force (*F*_*i*_), the indentation (Δ*z*_*i*_) is calculated as a difference between *z*_*xi*_ positions

3 $$ \varDelta {z}_i={z}_{2i}-{z}_{1i} $$

where *z*_*i*_ = *F*_*i*_ / *k*_cantilever_. As a result, the indentation curve is derived (Fig. 2b). Such a curve reflects the mechanical response of the studied sample to the applied load force, and it is characteristic for a given material.

## The Young’s Modulus Determination

Most commonly, Young’s modulus is evaluated in the frame of the Hertz contact mechanics [19] which describes the indentation of two purely elastic spheres. The model can be extended into a case when a sphere indents an infinitely thick, elastic half-space. A single cell cannot be treated as a thick and elastic half-space due to its internal structure composed of materials characterized by distinct mechanical properties. A cell cytoskeleton is built of stiff fibres of elasticity in the range of hundreds of megapascals or even few gigapascals that are surrounded by a viscous cytoplasm. Despite the ongoing development in modelling of mechanical properties of single cells, the Hertz contact theory is still dominant in the analysis of elasticity of single cells. It has been developed by Sneddon [20], introducing axisymmetric shapes of the indenter (i.e. spherical, paraboloidal, conical and flat-ended ones) into relations between the load force and the indentation depth. The direct use of these relations requires an assumption of the shape of the AFM probing tip (usually, it is a four-sided pyramid). This shape is typically approximated either by a cone or by a paraboloid or pyramid, leading to the following equations:

4 $$ \mathrm{Cone}:\kern0.5em F\left(\varDelta z\right)=\frac{2\cdot \tan \alpha \cdot E^{\prime }}{\pi}\cdot {\left(\varDelta z\right)}^2 $$

5 $$ \mathrm{Paraboloid}:\kern0.5em F\left(\varDelta z\right)=\frac{4\cdot \sqrt{R}\cdot E^{\prime }}{3}\cdot {\left(\varDelta z\right)}^{\frac{3}{2}} $$

6 $$ \mathrm{Pyramid}:\kern0.5em F\left(\varDelta z\right)=\frac{ \tan \alpha \cdot E^{\prime }}{\sqrt{2}}\cdot {\left(\varDelta z\right)}^2 $$

where *F* is the load force, Δ*z* is the indentation depth, *α* is the opening angle of the cone and *R* is the radius of the curvature of the AFM probing tip. The approximation of paraboloidal tip is used when spheres are used as probes; however, it is valid for indentations that are smaller than the sphere radius.

The *E*′ is the reduced Young’s modulus of a sample, described by the following relation:

7 $$ \frac{1}{E^{\prime }}=\frac{\left(1-{\mu}_{\mathrm{tip}}^2\right)}{E_{\mathrm{tip}}}+\frac{\left(1-{\mu}_{\mathrm{cell}}^2\right)}{E_{\mathrm{cell}}} $$

where *μ*_tip_ and *μ*_sample_ are the Poisson ratios representing the compressibility of the tip and a sample. It ranges from 0 to 0.5. For living cells, an elastic modulus is much smaller than Young’s modulus of the probing tip [3, 21], i.e. *E*_cell_ < < *E*_tip_; thus, the reduced Young’s modulus can be written as

8 $$ E^{\prime }=\frac{E_{\mathrm{cell}}}{\left(1-{\mu}_{\mathrm{cell}}^2\right)} $$

The exact value of *μ*_cell_ is unknown and difficult to determine. However, its value can be assumed to be 0.5, since cells can be treated as an incompressible material.

**Fig. 3:**
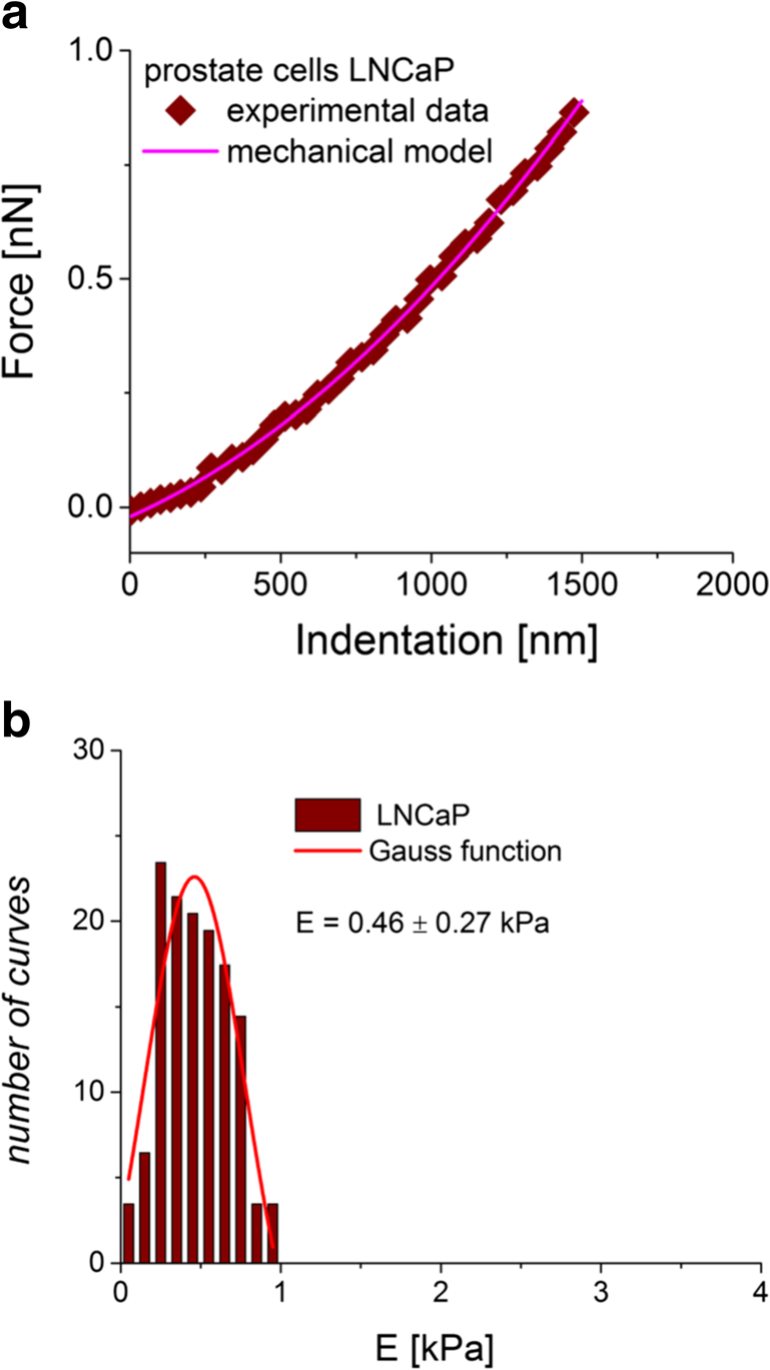
**a** The determined force versus indentation curve (*diamonds*) with a *line* being the fit of the mechanical Hertz model. **b** The final determination of Young’s modulus from the Gaussian function fit. The *centre* denotes the mean, while the half width taken at half height is attributed to standard deviation

During the analysis of the force–indentation curves, the fitted function is assumed to take a form of the power law *y* = *a* · *x*^*b*^, where the *b* value depends on the assumed shape of the intending AFM tip. The resulting fit very often follows the quadratic function (Fig. 3a), but this is not always the case. Sometimes, force–indentation curves are better described when *b* equals 1.5. Thus, to choose which model fits better, the goodness of fit, *χ*^2^, can be employed.

The final Young’s modulus is calculated, taking into account all values obtained from a whole set of force versus indentation curves. The resulted distribution is fitted with the Gauss function (Fig. 3b). The centre of the distribution denotes the mean value, while its half width taken at half height (HWHH) approximates a standard deviation. This is true that, for symmetric histograms, the non-symmetric ones require to apply another approaches like, for example, the use of the lognormal distribution [22].

The use of the Hertz–Sneddon model to quantify the elasticity of single cells is quite often discussed in terms of its applicability and appropriate experimental conditions. There are several issues, and the most important is the fact that indentation depth is not measured but calculated by subtracting the two curves measured on stiff and compliant surfaces. The stiff surface is usually the glass, serving as the substrate for studied cells; thus, two small deflections recorded for stiff surface could be burdened by impurities present on a surface on which cells are cultured, even though cells are far away of the chosen location. These impurities may stem, i.e. from adsorption of culture medium components. Impurities may decrease the slope of the reference, *stiff* curve, leading to smaller indentation values. Another source of potential trouble is the choice of cantilever. It is obvious that cantilever spring constant should be comparable with the stiffness of a cell (typically, its value ranges from 0.01 to 0.5 N/m [3, 6, 8, 17, 23–32]), but it is not the only parameter to be verified. The majority of cantilevers possess various pyramidal shapes characterized by distinct geometrical dimensions. When a small contact area will be combined with a large cantilever spring constant, a high pressure arises within the contact surface area of the probing tip and surface which can lead to cell surface damages. Moreover, the approximation of the pyramidal shape by already resolved indenter geometries that used the Hertz–Sneddon model (paraboloid, sphere, cone) can introduce additional uncertainty in modulus determination. Table 1 presents the brief summary of the AFM-based elasticity measurements carried out with the aim to distinguish cancerous cells from reference or healthy ones.

**Table 1:** Summary of the AFM-based elasticity experiments carried out for distinct cancerous cells

Cell tissue type	Modulus ratio (normal/cancer)	Cantilever	Tip shape/approximation for model	Reference
Bladder				
HCV29/Hu456	12	V-shaped	Pyramid/paraboloid	[3]
HCV29/T24	32	0.05–0.1 N/m		
HCV29/HTB9	5	V-shaped 0.01 N/m	Pyramid/cone	[23]
SV-HUC-1/MGH-U1	12	MSCT 0.01 N/m	Pyramid/not specified	[24]
Prostate				
BPH/LNCaP	9	V-shaped	Not specified/pyramid	[5]
BPH/PC-3	2	0.06 N/m		
PZHPV-7/LNCaP	6.8	V-shaped	Pyramid/cone	[17]
PZHPV-7/Du145	2.3	0.01 N/m		
PZHPV-7/PC-3	1.6			
Vero/Du145	2.2	CSG11	Sphere 9 μm/paraboloid	[25]
Breast				
MCF-10A/MCF-7	1.4–1.8	V-shaped 0.01 N/m	Sphere 4.5 μm/paraboloid	[26]
184A/T47D	1.9	V-shaped	Pyramid/cone	[17]
184A/MCF7	1.8	0.01 N/m		
HBL-100/MDA-MB-231	1.7	DNP	Pyramid/cone	[27]
HBL-100/MCF-7	1.1	0.35 N/m		
MCF-10A/MDA-MB-23	2.2	V-shaped 0.02 N/m	Pyramid/paraboloid	[28]
Cervix				
Normal/cancer primary cells	0.7	V-shaped	Sphere 5 μm/paraboloid	[29]
CRL2614/CaSki	2.8–3.8	V-shaped 0.06 N/m	Pyramid/cone	[30]
HeLa/End1(E6E7)	0.5	V-shaped 0.03 N/m	Pyramid/cone	[31]
Thyroid				
Primary thyroid cells	3–5	V-shaped	Pyramid/cone	[8]
S748/carcinoma cell S277		0.01 N/m		
Ovary				
IOSE/HEY	2.8	MCST	Sphere 4.7 μm/paraboloid	[6]
IOSE/HEY A8	5.0	AUHW		
IOSE/OVCAR-3	4.3	0.03 N/m		
IOSE/OVCAR-4	2.2			
Chondrocytes				
Chondrosarcoma cells				
FS090 (grade II)/JJ102 (myxoid chondrosarcoma)	3.6	V-shaped	Sphere 4.7 μm/thin-layer Hertz model	[32]
FS090 (grade II)/105KC (myxoid chondrosarcoma)	1.6			

Those elastic properties were determined based on the Hertz–Sneddon model using various shape approximations of the indenting AFM probe

The data presented in Table 1 cannot deliver the easy answer of which type of the cantilever (in particular, which geometry of the probing tip) should be chosen for elasticity measurements of living cells. The use of spherical probes in elasticity measurements delivers results showing smaller detectable differences in the elasticity between normal and cancerous cells [25, 26, 29, 33]. Still, cancerous cells are better detectable in measurements where pyramidal probes are used. This can originate from the lack of good calibration of the cantilever spring constant or from the way of gluing a sphere to a tipless cantilever. The only study of ovarian cancer shows the elasticity measurements with reliable sensitivity that could be used in clinics [6]. On the other hand, the results obtained with the use of typical pyramidal probes of various dimensions and types indicate that differences between normal and cancerous cells are cancer type specific and that the highest standards in the design of AFM probes for clinical application are still not fully met. In summary, it seems that, for a moment, the better differentiation between normal and cancerous cells can be obtained using standard pyramidal cantilevers, which can deliver relative modulus values. Their use in cancer cell detection should be always accompanied by reference measurements. To obtain absolute, true modulus values, spherical probes are undoubtedly better, since they provide better agreement with theoretical expectations [34].

## Data Verification: a Link Between Cellular Stiffness and Actin Cytoskeleton

**Fig. 4:**
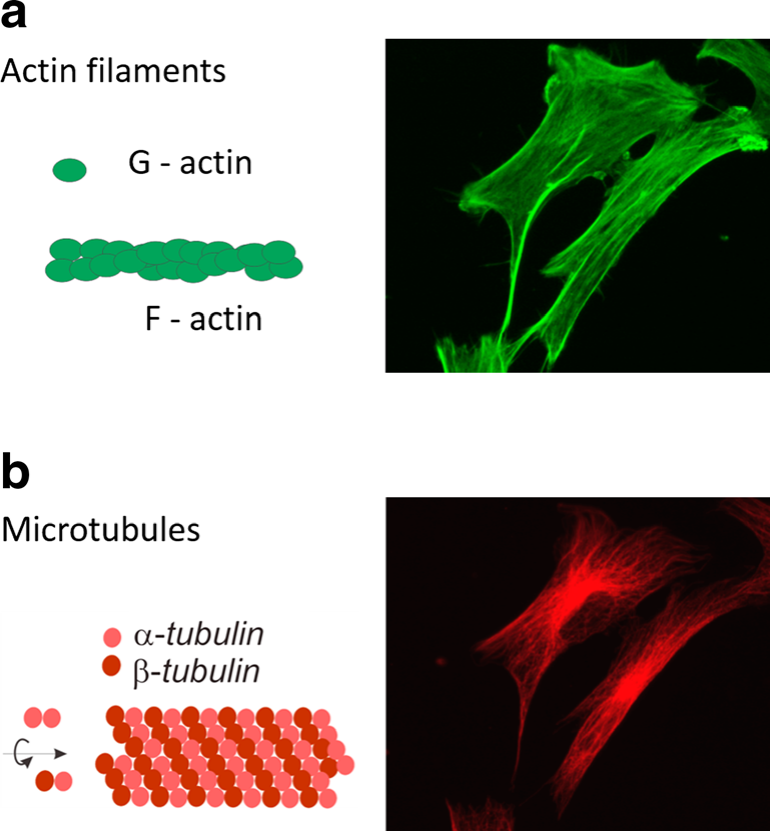
Fluorescent images presenting the distribution of two main structural elements of a cytoskeleton inside the cell: **a** actin filaments and **b** microtubules. Images are accompanied by a schematic illustration of single filaments

Various studies brought data demonstrating that cellular elasticity is strongly linked with the cell cytoskeleton. A cell cytoskeleton is a dynamic network of three main fibrous structures, namely actin filaments (composed of F-actin, i.e. a polymerized form of G-actin molecules, Fig. 4a), microtubules (composed of two isoforms of tubulin, i.e. α- and β-tubulins, Fig. 4b) and intermediate filaments. Each type of filaments is specifically distributed inside the cell. The F-actin forms both short actin filaments and stress fibres (bundles of single filaments). The former are mostly located beneath a cell membrane, while the latter span over a whole cell body. The microtubules start from the centre located nearby the nucleus and end anchored in the cell membrane (the corresponding fluorescent images are presented in Fig. 4).

**Fig. 5:**
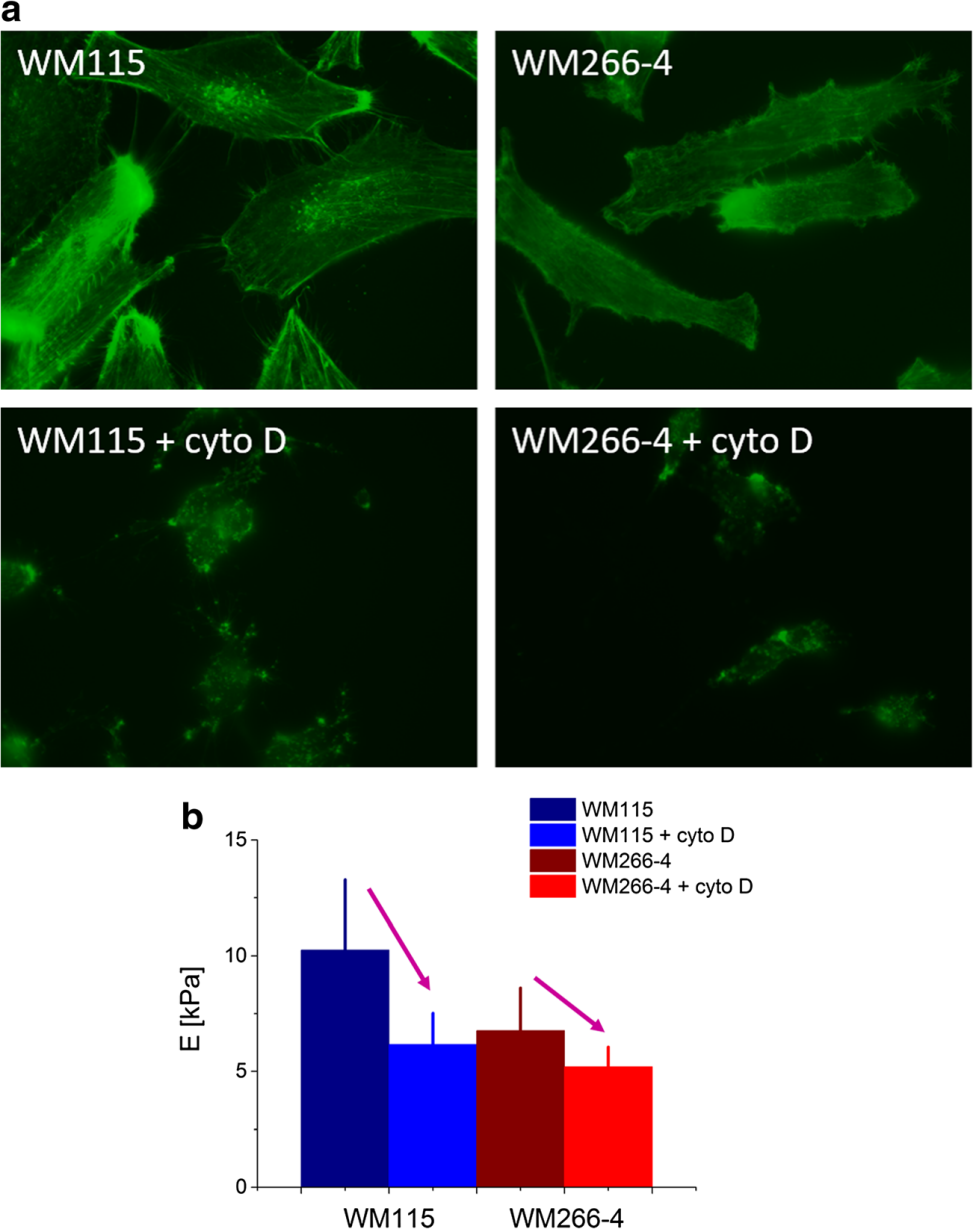
The cytochalasin D effect on **a** organization of actin filaments in melanoma cells as presented in fluorescent images and **b** elasticity of single cells

All fibrous cytoskeletal structures interact with each other, providing the mechanical stability of cells. Various measurements carried out on single cells delivered data showing that, depending on the cell type, either actin filaments or microtubules dominate in a mechanical response of cells measured by AFM. To demonstrate which type of cytoskeleton filaments dominates in the mechanical response, living cells are incubated with so-called cytoskeletal drugs that influence the stability of cytoskeletal filaments [23, 35–37]. The most common cytoskeletal drug used is the cytochalasin D, which depolymerizes actin filaments that can be observed by either (a) the fluorescent microscopy (Fig. 5a) or elasticity measurements (Fig. 5b).

Melanoma cell treatment with a 5 μM concentration of cytochalasin D for 30 min causes depolymerization of long actin fibres, previously visible in non-treated cells. This results in a significant drop of Young’s modulus denoting the increase of cellular deformability. Such experiments demonstrate that the elastic properties of living cells are closely related to actin filaments. In early studies [23, 35, 36], only their organization inside the cell was considered. In particular, some results showed that the presence of stress fibres (i.e. bundles of single actin filaments) influences strongly the elasticity of cells [38] (i.e. the larger modulus values) like the case of bladder cancer [17]. Recently, some data show that not filament organization alone is responsible for a given state of cellular elasticity. Also, the total amount of actin is related to cellular stiffness [25, 39]. This may lead to a conclusion that the density of actin is more related to alterations in cellular elasticity induced by oncogenic transformation than the organization of actin filaments alone. Some studies demonstrated that mechanical properties of colon cancerous cells depend on the ratio of actin filaments to microtubule content [10]. The disrupting and/or stabilizing effect of cytoskeletal filaments is dependent on cell type, chemical compound and filament type. Thus, the effect of cellular deformability can be manifested in both higher and lower deformability (cells become softer or more rigid, respectively).

## Relativeness of Young’s Modulus

The AFM-based determination of the absolute value of Young’s modulus is not an easy task due to effects stemming from various factors. They can be linked with (1) the uncertainties related to the applied methodology for the cantilever choice, calibration of its spring constant and photodetector sensitivity; (2) the experimental conditions provided by the AFM such as load speed, place of poking, number of force curves recorded at one place, location of the measurements on cell surface and the presence of the stiff substrate below the investigated cells; and (3) the way of data analysis (especially the determination of the point of contact between the indenting AFM tip and cell surface, range of indentation depth or load force), including also the mechanical models applied to describe the elasticity of living cells. The other important groups of factors influencing the mechanics of cells are those directly influencing the cellular properties, such as culture conditions (buffer composition), the density of cell confluence on a substrate, the number of passages, the day of measurement after the passage and physical and chemical substrate surface properties influencing cell behaviour.

**Fig. 6:**
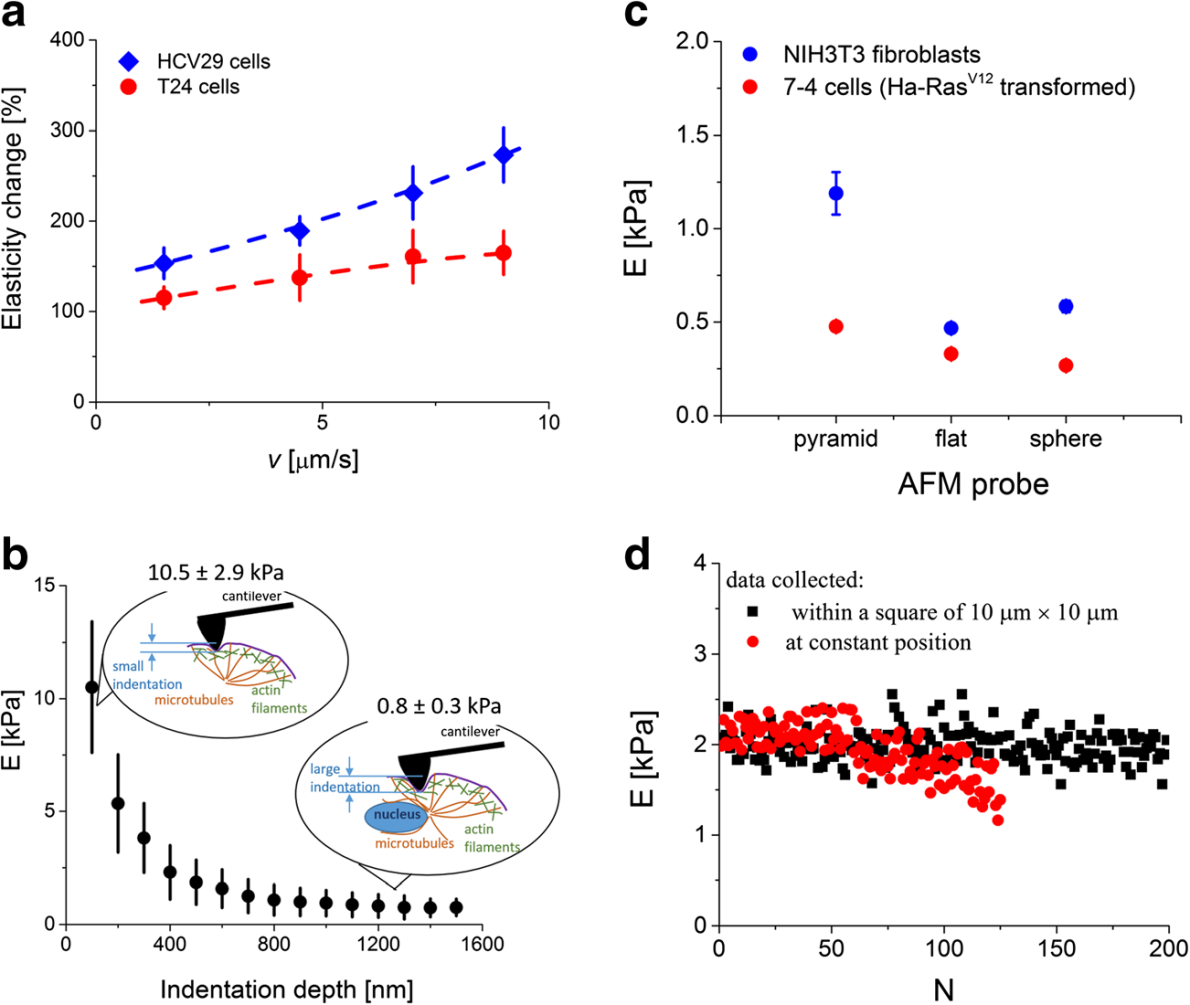
The single cell elasticity changes observed as **a** a function of load speed, **b** indentation depth, **c** probing tip geometry (calculated based on [39]) and **d** in case of prolonged poking carried out both at the single location and within a grid of 10 μm on a single cell (*N* denotes the number of consecutive poking)

The appropriate calibration of the cantilever spring constant and photodetector sensitivity is vital, since the errors introduced by their variations influence strongly the accuracy of the elastic modulus determination. The experimental conditions provided by the AFM technique alone are mainly linked with the way how the AFM-based force spectroscopy is realized in a particular device. This include, among others, load speed that is related to loading rate, indentation depth, probing tip geometry and the place and number of force curves recorded (Fig. 6).

Several studies reported so far have shown that Young’s modulus is a load rate-dependent quantity. In the AFM, it is not possible to directly control the load rate value. Its value can be indirectly modified by changing the speed of indentation (referred here as *load speed*). This has been demonstrated, for example, for breast [26], bladder [7], prostate [17] and red blood cells [40]. Noteworthy, these results show that normal cells are more sensitive to the load rate (the elasticity change is larger) as compared to cancerous ones (Fig. 6a). The lack of homogeneity within a structure of single cells and their viscoelastic nature can result in variations of the elasticity modulus that are indentation depth dependent (Fig. 6b). In such case, Young’s modulus reflects the mechanical response originating from various cellular structures [41]. For smaller indentation depths, the elasticity of single cells is dominated by the mechanical response of filamentous network of actin filaments. Thus, the heterogeneity of the elastic modulus distributions reveals the distinct and irregular organization of actin filaments lying beneath the cell membrane within a range of indentation up to 200 nm (this can be also verified by cytochalasin D treatment). The choice of larger indentation depths enables probing of cellular regions rich in all cytoskeleton elements (i.e. actin filaments, microtubules and intermediate filaments). In such case, the overall elasticity of the whole cell can be obtained. The correct choice of indentation depth can be essential for identification of pathologically changed cells occurring, for example, in case of cancer where distinct organization of cell cytoskeleton is expected [9].

In the AFM, the cell surface is probed by means of cantilever with the probing tip mounted at its free end. The relativeness of Young’s modulus manifests in the distinct elasticity generated in response to various geometrical properties of the indenting tip. The relation between Young’s modulus and the geometry of the probing tip is demonstrated in Fig. 6c, which presents the modulus values calculated for three types of indenting probes: pyramidal, flat-ended and spherical ones [39]. One can see that the use of pyramidal tip delivers larger elastic modulus values as compared to those obtained for flat-ended or spherical (bead diameter of 5 μm) indenters. The effect is also dependent on cell type, as observed from the comparison between NIH3T3 and Ha-Ras^V12^ oncogene-transformed fibroblasts (Fig. 6c).

The demand of high statistics requires a prolonged poking of every single cell that can lead either to a damage of cell membrane or to remodelling of actin cytoskeleton (Fig. 6d). As a consequence, alterations in cellular deformability are expected. They can manifest in a sudden change (a drop or an increase) of Young’s modulus. Exemplary data, included in Fig. 6d, presents the Young’s modulus calculated from force curves recorded during poking at a predefined, constant position. The measured moduli, randomly distributed around the mean value of 1.20 ± 0.28 kPa, start to decrease with time, indicating a change in the elastic modulus and thereby alterations in the organization of actin cytoskeleton. More stable data can be obtained by setting a square area, e.g. 10 μm × 10 μm, within which force curves are acquired (blue dots in Fig. 6d). Here, the prolonged poking does not induce/generate such clearly visible remodelling of actin cytoskeleton as compared to poking at a single location.

The above-mentioned factors influencing the obtained value of Young’s modulus are not the only one. A separate group is related to the assumptions required for the Hertz contact mechanics, a widely applied theoretical model used for the quantification of deformability of living cells. These assumptions are only partially fulfilled since they describe the cell as an isotropic, purely elastic material that can be approximated by an infinitely thick half-space. Additionally, the Hertz contact mechanics can be used only under the assumption of no adhesion forces present within the contact area between the probing tip and the cell area.

**Fig. 7:**
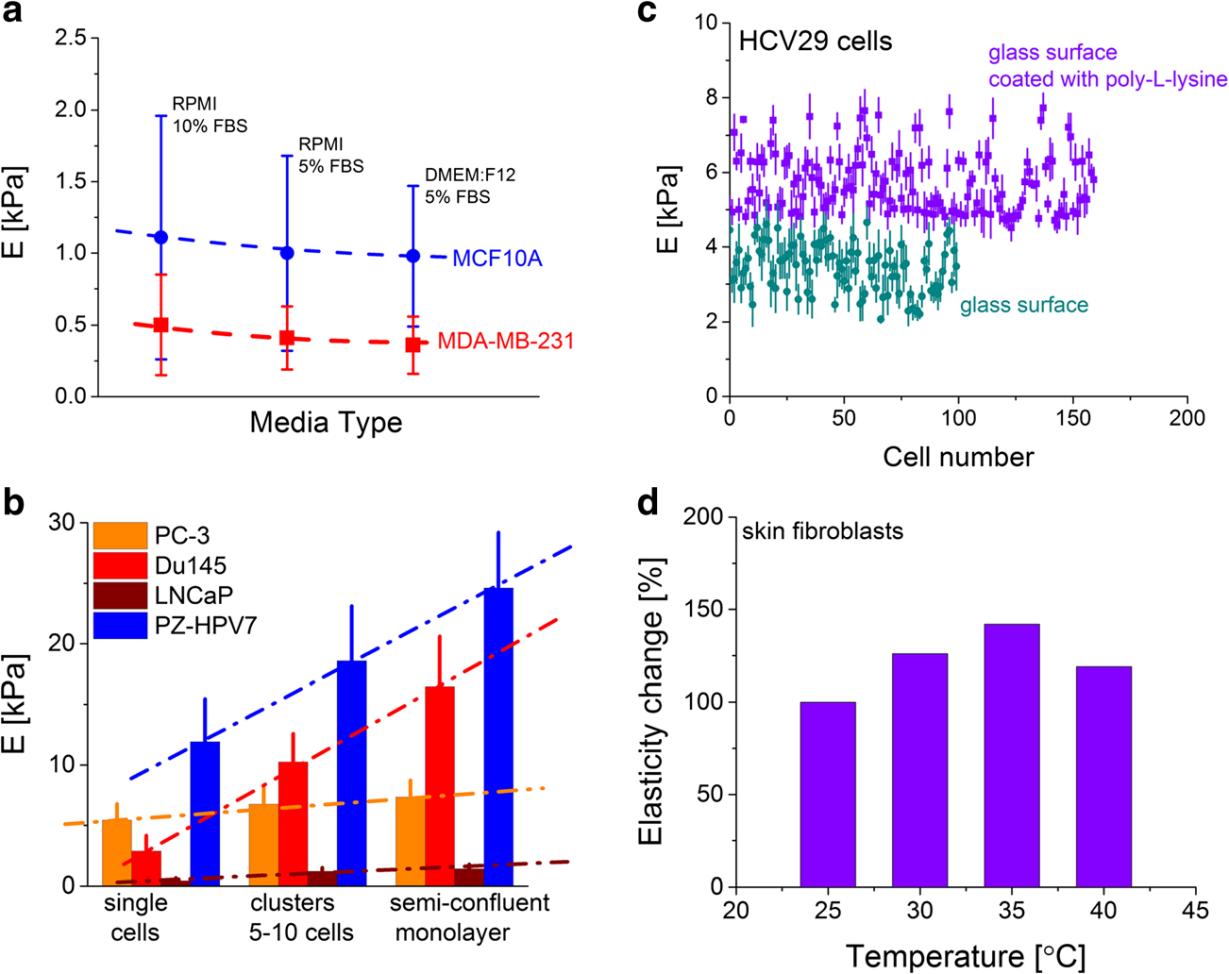
Examples of cell-related factors influencing the Young’s modulus value of living cells. The effect of **a** medium composition (prepared using the data published in [28]), **b** cellular density, **c** surface chemical and physical properties and **d** temperature on the elasticity of living cells

Various researchers demonstrated that cells respond to changing environmental conditions of both chemical and physical origins. This leads to cell-related variations, such as medium composition, density of cells, temperature and surface chemical and physical properties (Fig. 7). Usually, this source of variation origin is more significant for elasticity determination than discrepancy arising from the applied calibration, the way of the data acquisition and analysis.

Probably, the most important one is a medium composition, since it may affect the results of any comparison between various cell lines. An example is presented in Fig. 7a showing the results for two breast cell lines, namely non-malignant (MCF10A) and metastatic (MDA-MB-231) ones [28]. Only the change of the foetal bovine serum concentration form 10 to 5 % decreases Young’s modulus for about 15–20 %. The alterations of the basic medium from RPMI 1640 to DMEM/F12 resulted in the increase of cellular deformability (Young’s modulus decreases) independent of the cell type. However, it is more dominant for non-malignant MCF10A breast cells. These results strongly suggest the use of similar medium composition when comparison of mechanical properties is the aim (or serious consideration of the effect of medium composition if it is not possible to use the same culture medium). Simultaneously, such studies could be accompanied by fluorescent images of actin cytoskeleton, in order to verify whether culture medium composition changes the actin filament organization. There are more findings showing that the elasticity of living cells is generally not constant. Cells change in response to surrounding environment. Thus, it is obvious that the neighbouring cells influence the mechanics of the particular, studied cell. When the elasticity is investigated as a function of the cellular density, depending on the cell type, various relations are expected. For example, for prostate cells (Fig. 7b), the increase of Young’s modulus can be observed. The magnitude of changes is cell type dependent. Among four studied cell lines, two of them, i.e. PZHPV-7 (derived from a healthy gland) and Du145 (brain metastasis), respond significantly to the presence of neighbouring cells, while for two other cell lines, i.e. LNCaP (lymph node metastasis) and PC-3 (bone metastasis), Young’s modulus changes slowly.

The mechanical response of cells cultured on a stiff, non-deformable surface depends on chemical properties of the substrate surface used for cell growth. In one of the first paper, the effect of substrate properties was studied in the context of surface suitability in tissue engineering. Osteoblasts cultured on various substrates (CoCr, Ti, TiV, glass and tissue culture polystyrene) revealed the elasticity range from 2 Pa (observed for CoCr and TiV substrates) to 9 kPa for Ti surface. The latter modulus was comparable for that obtained for osteoblasts cultured on polystyrene surface [42]. Simple modification of substrate surface, by coating it with poly-l-lysine, a common compound enhancing cell adhesion, influences the modulus value as it is shown in Fig. 7c. For human bladder cells (HCV29—non-malignant cancerous cell of the ureter), covering the glass surface with poly-l-lysine produced a 1.5-fold increase of Young’s modulus.

Cells are cultured at a temperature of 37 °C that resembles physiological conditions. The majority of papers reported that the elasticity measurements were carried out at room temperature (usually between 20 and 22 °C). Figure 7d presents the elasticity modulus change for skin fibroblasts (CCL-110). Initially, up to 35 °C, its value raises to 142 % of the value determined for cells measured at 25 °C. The further temperature increase to 40 °C manifests in a drop of elasticity that is probably linked with the strong reorganization of actin cytoskeleton, leading to partial protein denaturation. These data are consistent with that reported by Chio et al. [39], where NIH3T3 fibroblasts seemed to be unaffected within the temperature range of 31–37 °C, whereas the increase to 43 °C caused a sudden drop of the modulus value. For the seven to four cells, a modulus maximum at 37 °C was observed. Also, intuitively, one can expect that the density of cells influences the mechanics of single cells if cells attach to each other. Such cellular interconnections involve the formation of additional actin filaments, leading to their mechanical strengthening. The effect of cell confluency on elasticity has been observed for normal Vero [43] and HMEC [44] cells. The former results showed that cells in the monolayer had lower Young’s modulus (of about 1.4–1.7 times), while the latter increased their stiffness with density of cells. Studies on parallel immortal, tumorigenic and metastatic cells revealed a distinct relation on the surrounding environment. The stiffness of immortal and metastatic cells was unaffected by the presence of neighbouring cells, whereas tumorigenic ones seemed to become slightly softer [44].

All sources of errors mentioned within this section lead to the conclusion that the determination of the absolute Young’s modulus is very difficult and may raise doubts in the usefulness of the AFM in the quantification of the elasticity of living cells. However, first of all, the exact knowledge of the absolute Young’s modulus is not always needed. It can be overcome by comparing the results with reference cells, measured in the same experimental conditions. In fact, multiple research papers published so far prove that, despite the various uncertainty sources, the relation between normal (healthy or reference) and cancerous cells is preserved. Nevertheless, all gathered evidence, demonstrating that elastic modulus can be used as a biomarker of cancer, demands a high reproducibility of elasticity measurements, enabling verification within various laboratories. This asks for standardization protocols that could be widely applied, and that could deliver a reference set of samples produced independently in various laboratories. One of the first approaches tackling this issue has been proposed in 2015 by Demichelis et al. [45]. In this work, the polydimethylsiloxane (PDMS) material has been used to produce samples with the stiffness range of 50–5000 kPa. The AFM-based indentation measurements showed the stability over time in the elastic modulus range from 500 to 5000 kPa, demonstrating modulus reproducibility at the level of 4 %. The only drawback is that the elasticity of living cells is few times lower (up to 100 kPa) [18] that leads to conclusion that some softer, highly homogenous and stable material is strongly needed. Next, it could be simultaneously used to mimic mechanical properties of living cells and to serve as a reference sample.

## Comparing Properties of Single Cells

**Fig. 8:**
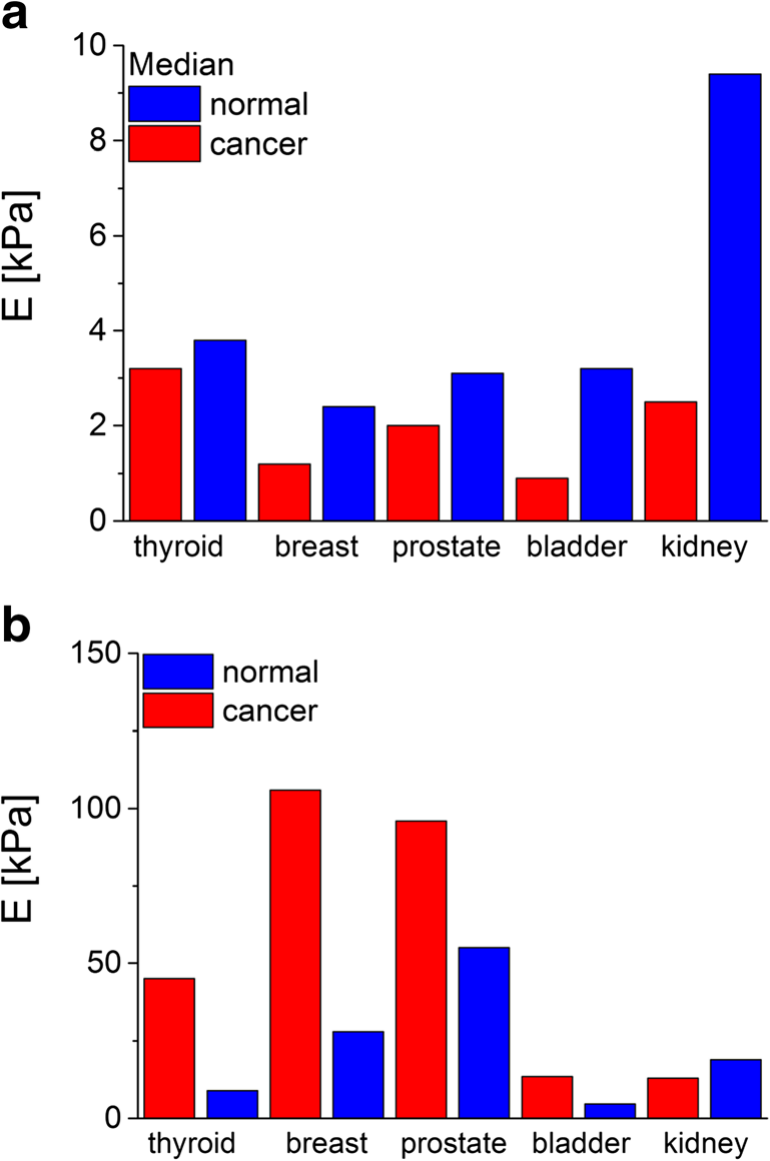
The comparison of Young’s modulus determined for various cancers at **a** single cell and **b** tissue levels. These images were prepared based on data included in [3, 7, 8, 17, 46]

The basic application of the AFM technique in the determination of mechanical properties of living cancerous cells is focused on the comparison between the reference (normal, healthy or originated from earlier stages of cancer progression) and cancerous ones. This has been shown for various cancer types including, for example, thyroid [8], breast [7, 27], prostate [5, 17], bladder [3] and kidney [46] cancers (Fig. 8a, prepared based on data reported in [3, 7, 8, 17, 46]).

The determination of single cell deformability indicates the potential use of Young’s modulus as a quantitative biomarker of cancer-related changes. Thus, it is desirable to find its correlation with histological grades used to classify the cancers as it has been reported for ovarian cancer [6]. Analogous to other cancers, the non-malignant immortalized ovarian surface epithelial (IOSE) cells show larger Young’s modulus (2.472 ± 2.048 kPa) than two other cell types derived from the same ovarian cancer cell lines, i.e. HEY (0.884 ± 0.529 kPa) and HEY A8 (0.494 ± 0.222 kPa) ones. The migratory and invasive properties of both studied HEY and HEY A8 cells display the largest invasive and migratory activity for HEY A8 cells and the lowest for IOSE control cells, indicating that cellular stiffness is inversely correlated with the indicators of metastatic potential (migration and invasiveness).

Beyond doubt, one can state that single cancer cells are more deformable as compared to their normal counterparts. This seems to be in contradiction with a macroscopic image observed by medicine, in which cancers are sensed by palpation as a solid, stiff mass. Furthermore, Young’s modulus of tissue samples taken from solid tumours is often larger as compared to that of normal tissue samples (Fig. 8b, prepared based on data reported in [46, 47]). The explanation of such situation is relatively simple: Methods used to determine the modulus in tissue samples are macroscopic (usually, a sample of mm or cm size is needed); thus, the recorded elasticity reveals the overall mechanical response of a whole sample volume without separation on particular constituents (single cells or ECM). The larger deformability of cancerous cells probed by the AFM reflects their possibility to enhanced movement and migration, linked with the increased invasiveness like in the case of ovarian cancer [6]. However, the relation is not obvious for other cancer types. The stiffening of tumours observed in macroscopic measurements indicates the importance of ECM components in cancer progression [48]. It seems to be confirmed by the measurements of tissue elasticity carried out by atomic force microscopy, in which very broad distributions of Young’s modulus are observed [17, 51, 52]. For such heterogeneous samples like tissue, smaller modulus values can be attributed to the elasticity of single cells while the larger ones correspond to extracellular matrix components, like collagen fibres forming dense deposits in various cancers [50, 52].

## Single Cell Elasticity as an Indicator for Change Monitoring

**Fig. 9:**
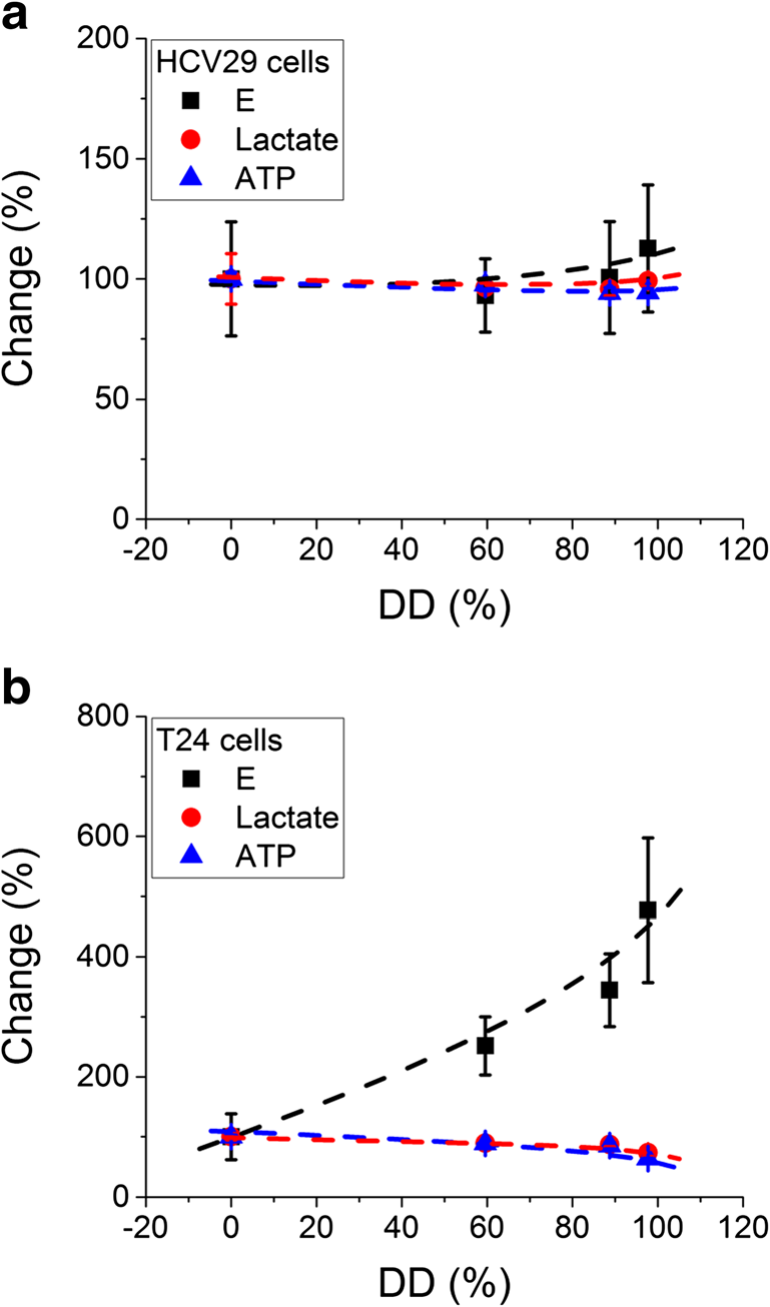
**a**, **b** The effect of chitosans on the elasticity and glycolytic activity of non-malignant HCV29 and cancerous T24 cells (*DD* denotes the deacetylation degree expressed in %). The glycolytic activity was measured as lactate and ATP levels. All data were normalized to the control value obtained for non-treated cells (data were prepared based on [54])

The determination of cancer cell elasticity and its comparison with normal cells opened the wide spectrum of applications using the elasticity as an indicator for direct monitoring of changes induced by various factors interacting with cells. The obvious application is to trace how distinct compounds added to the medium surrounding the cell induce alterations in elastic properties. Among several systems studied, the correlation between the glycolytic activity and the single cell deformability in human bladder cancers nicely demonstrates the functionality of the AFM [53]. In this research, both the levels of glycolytic molecules (ATP and lactase are the products of glycolysis process) were measured in parallel with the deformability of single cells as a function of chitosan characterized by various deacetylation degrees (defined as the ratio between the number of amino groups and the total number of both amino and acyl groups). The results showed a decrease in ATP and lactate levels, indicating the inhibition of glycolysis that was accompanied by the strong decrease in cellular deformability (the elastic modulus increased up to 4 times) in cancerous cells but not in non-malignant cancer cells (Fig. 9).

The metabolism of most cancerous cells shows larger activity that is usually linked with the overexpression of the glycolytic enzymes like pyruvate kinase type M2 [54, 55] that can be associated with the cell cytoskeleton. Thus, the detachment of these enzymes can lead to the decrease in the glycolic activity and, in parallel, to reorganization of cell cytoskeleton realized by the rearrangements in both actin and microtubule networks [56]. The deacetylation degree (DD) is attributed to the charge, which is proportional to the amount of amino groups (positively charged in water solutions). Since the high molecular mass of chitosans disables these molecules to enter the cell, their interaction with cells is restricted to membrane surface only, probably, by binding the positively charged molecules of chitosan to the negatively charged membrane. Such mechanism is suggested to be the strongest when occurring between the chitosan with the highest deacetylation degree and the cell surface.

Noteworthy, the interaction with cancer cells seems to be more specific as compared to reference, non-malignant HCV29 cells. In these cells, the levels of lactate and ATP were almost similar, regardless of the deacetylation degree (i.e. charge) and only a weak decrease in cellular deformability was observed (Fig. 9a). Such a difference between HCV29 and T24 cells can be explained by the assumption of the smaller negative charge on the surface of the non-malignant cells, as compared to the latter ones. Therefore, the reference cells are less covered with positively charged chitosan and chitosan effect is much lower (Fig. 9b).

**Fig. 10:**
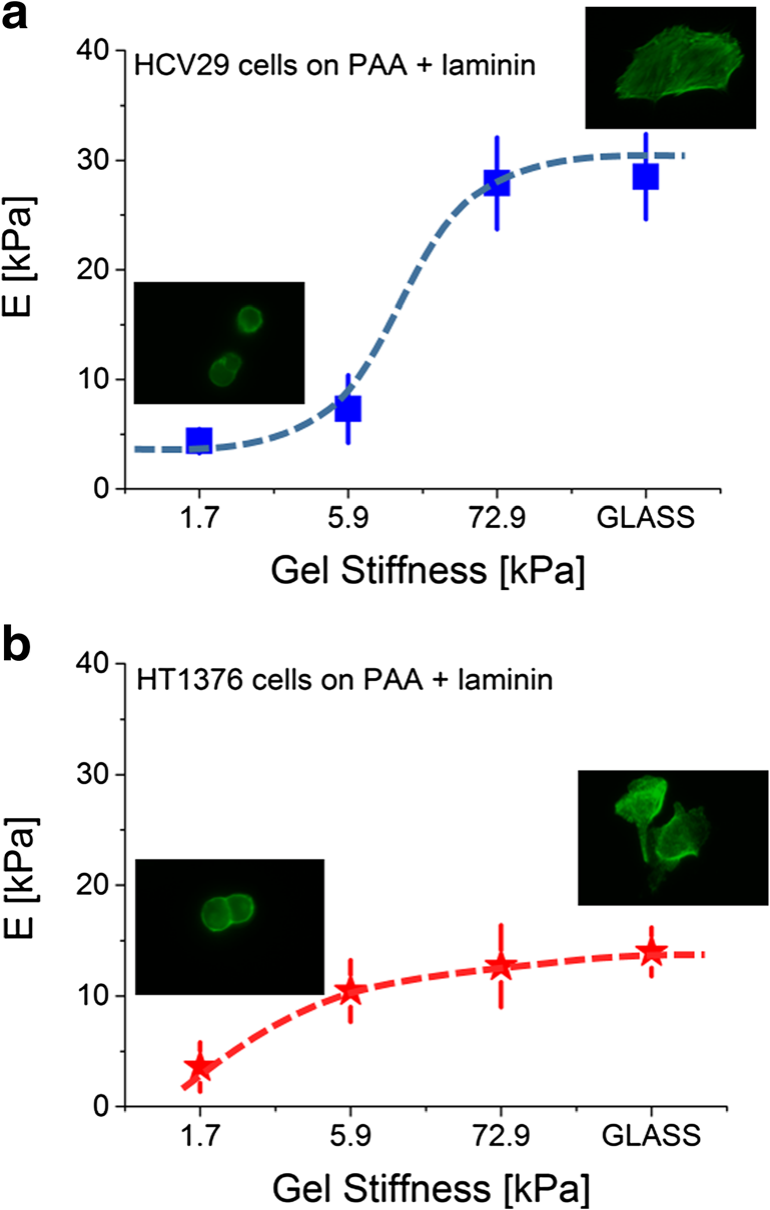
**a**, **b** Human bladder cancer cell (HT1376, transitional cell carcinoma) response to substrate stiffness/polyacrylamide gels covered with laminin compared to HCV29 non-malignant cancer cells of the ureter. Young’s modulus was calculated for the indentation depth of 200 nm

The other examples of using the cellular deformability as a biomarker of induced changes are studies on mechanical response of living cells to the surrounding environment. Over the past years, a great effort has been made to understand the influence of substrate stiffness on the behaviour of living cells. Their response is thought to play an important role in both cell functioning and disease development and progression [49]. The elastic properties of various tissues in living organisms vary from few pascals for very soft tissues like brain to tens of kilopascals in muscles and even to megapascals for some cartilages [47]. To study the effect of the mechanical properties on single cells, hydrogels, such as polyacrylamide or collagen ones, are used to mimic the cellular environment with stiffness within the range from 10 Pa to hundreds of kilopascals. The effect of substrate stiffness on living cell properties, growth and differentiation has been demonstrated mostly by normal cells. However, recently, several studies have shown the influence of substrate stiffness on cancerous cell properties [56–60]. As an example, the mechanical response to various substrate stiffness observed for human cancer bladder cells is presented in Fig. 10.

Bladder cancer cells shows altered mechanosensitivity manifested in distinct mechanical response to various substrate stiffness, as compared to HCV29 cells. The altered mechanosensitivity of cancerous cells is attributed to their mesenchymal phenotype acquired during cancer progression. When cells are cultured on a stiff glass substrate, bladder cancer cells are softer than non-malignant ones (14.0 ± 2.2 versus 28.5 ± 3.9 kPa, respectively; Young’s modulus was calculated for the indentation depth of 200 nm, assuming the dominant role of substrate stiffness in cellular mechanics). In both cell lines, the elastic modulus changes as a function of polyacrylamide stiffness but the character of the response is different depending on the cell type. The altered response of cancer cells to substrate stiffness suggests that the lower magnitude of the decrease in cellular deformability might contribute to its augmentation.

## Considering the Viscous Nature of Living Cells

Various techniques, such as optical [61] or magnetic [62] tweezers, delivered data proving the viscoelastic nature of living cells, depending on the time scale of force application. Thus, mechanical properties of cells can be described in terms of both their elastic (quantified by Young’s modulus) and dissipative (viscous) components. The AFM working in a standard elasticity measurement mode delivers only the elasticity of cells. There is increasing but still not sufficient information on frequency-dependent mechanical properties of living cells. In early work, Shroff et al. studied dynamic changes of quiescent and contracting rat atrial myocytes [63]. In this work, a 200-Hz sinusoidal perturbation was superimposed on the native cells. By recording the amplitude and phase of cantilever deflection, an absolute elastic modulus and the loss tangent were obtained, respectively. Later, other researchers applied a scanning probe-based microrheology approach to quantify the frequency-dependent viscoelastic behaviour of fibroblast cells. The results were compared to those recorded on polymer gel samples. Cells showed a viscoelastic signature that was quantified using an extended Hertz model introduced to measure the frequency-dependent storage and loss moduli [64]. The osculating tip in liquid conditions is influenced by various phenomena, such as viscous drag that is inversely dependent on the distance between the probing tip and sample surface [65]. These results showed that the storage and loss modulus increases are following a power law relation, while the loss modulus exhibits a steeper rise. Unfortunately, the comparison of viscoelastic properties of human alveolar (A549) and bronchial (BEAS-2B) epithelial cells was not possible. Both studied cell types showed a similar degree of viscoelastic properties [65]. In another study, biomechanical properties of JJ012, FS090 and 105KC chondrosarcoma cell lines were carried out to determine both elastic moduli and apparent viscosities by fitting the stress–relaxation data with the thin-layer elastic and viscoelastic models [66]. The findings showed that chondrosarcoma cells can be modelled by the derived thin-layer, viscoelastic model for stress–relaxation indentation. The mechanical properties of these cells, both elasticity and viscosity, showed a time-dependent behaviour. The most aggressive and invasive chondrosarcoma cell line, JJ012, revealed the lowest elastic moduli. Two other studied cell lines, FSC090 and 105KC, showed similar modulus values, but still, differences were large enough, enabling to distinguish between cell types (a similar trend was observed after 2 h and 2 days of culture). Apparent viscosity was significantly lower for the most aggressive chondrosarcoma cell lines measured after 2 h of culture, but it increased after 2 days of culture to the level of the other studied cell lines, making them indistinguishable. In the other studies [46], the viscosity of kidney cells was 69.6, 28.1 and 2.48 Pa for non-tumorigenic RC-124, carcinoma A-498 and adenocarcinoma ACHN cells, respectively. The large difference in cellular viscosity was accompanied by a distinct elastic modulus of 9.38, 7.41 and 2.48 kPa, correspondingly. One of the possible reasons of the relatively few data on dissipative properties of living cells is the lack of appropriate models that can be used to describe cellular elastic and viscoelastic properties based on AFM data. There were some attempts comparing the suitability of various theoretical approaches to describe mechanics of single cells that accounts for various factors influencing mechanics (e.g. [67]); however, still, there is no comparison showing whether and how cellular viscosity can be used as a biomarker of cancer-related changes.

## Conclusions

The AFM measurements of single cell deformability brought a novel approach that helps to understand the correlation between cell structure, mechanics and functioning. Despite the lack of the absolute value of Young’s modulus, the obtained relative change of the elastic modulus has been shown to be sufficient to describe alterations observed for cancerous cells. Furthermore, measurements of Young’s modulus, carried out on the single cellular level, can in the future help to determine the range of cytoskeleton changes, to allow their quantification, and to use them to describe the influence of drugs, the sensing of substrate stiffness, and to correlate the cellular deformability with the malignancy degree. The quantification of cellular deformability at a single cell level seems to help and to advance knowledge in various aspects, like cancer cell interactions with extracellular matrix, following the mechanism of their migration to distant places in the body and a formation of tumour metastatic sites.

The mechanical properties of single living cells have been recognized to be crucial in various diseases encompassing various sources of their origins (genetic modifications of cytoskeleton–membrane links like in muscular dystrophies, anaemias, hypertension, coronary and pulmonary diseases and cancer). Many studies demonstrated that cancerous cells are softer than cells from normal or non-malignant or even less-differentiated cancers. However, despite that, still, not many clinicians believe that it is possible to detect cancerous cell changes by mechanical properties. Usually, the relevance of elasticity measurements on single cells is questioned due to lack of a proper tissue environment provided in the experiments and/or due to the large structural complexity and heterogeneity of tumours.

The AFM strength stems from the high-resolution imaging and also from the ability to quantitatively characterize biophysical properties of single living cells. The ongoing technological development allows to carry out highly complex experiments where the AFM delivers unique information on cellular or molecular processes, not always accessible in other techniques. However, its use in clinical practice still needs more systematic studies on factors influencing mechanical properties of living cells. Elaborating them will deliver more understanding on cell biomechanics and also will bring AFM into consideration as a better detecting tool. By providing a deeper knowledge on cancer-related changes in various clinical materials, the AFM technique might significantly contribute to early proper diagnosis of cancer but its application in real clinical samples still requires a standard operational protocol to be established to make the comparison of results possible between different laboratories.
